# Obesity-Resistant Mice on a High-Fat Diet Display a Distinct Phenotype Linked to Enhanced Lipid Metabolism

**DOI:** 10.3390/nu16010171

**Published:** 2024-01-04

**Authors:** Fadia Milhem, Emily Skates, Mickey Wilson, Slavko Komarnytsky

**Affiliations:** 1 Plants for Human Health Institute, NC State University, 600 Laureate Way, Kannapolis, NC 28081, USA; fjmilhem@ncsu.edu (F.M.); eskates@ncsu.edu (E.S.); mlwilso8@ncsu.edu (M.W.); 2 Department of Food, Bioprocessing, and Nutrition Sciences, NC State University, 400 Dan Allen Drive, Raleigh, NC 27695, USA; 3 Department of Nutrition, University of Petra, 317 Airport Road, Amman 11196, Jordan

**Keywords:** dietary fats, energy substrate, body composition, metabolic rate, individual variability, personalized nutrition

## Abstract

Individually, metabolic variations can significantly influence predisposition to obesity in the form of the obesity-prone (super-responders) and obesity-resistant (non-responders) phenotypes in response to modern calorie-dense diets. In this study, C57BL/6J mice (n = 76) were randomly assigned to either a low-fat diet (LFD) or a high-fat diet (HFD) for 6 weeks, followed by selection of the normally obese (HFD), non-responders (NR), super-responders (SR), or super-responders switched back to the low-fat diet (SR-LFD) for an additional 8 weeks. SR mice showed the highest gains in body weight, lean and fat body mass, and total and free water, in part due to increased feed efficiency, despite having a respiratory exchange ratio (RER) similar to that of NR mice. A switch to the LFD was sufficient to revert most of the observed physiological changes in the SR-LFD mice; however, voluntary physical activity and exercise capacity did not return to the basal level. NR mice showed the highest food intake, lowest feed efficiency, increased oxygen consumption during the light (rest) cycle, increased physical activity during the dark (active) cycle, and increased heat production during both cycles. These variations were observed in the absence of changes in food intake and fecal parameters; however, NR fecal lipid content was lower, and the NR fecal microbiome profile was characterized by reduced abundance of *Actinobacteria*. Taken together, our findings suggest that NR mice showed an increased ability to metabolize excessive dietary fats in skeletal muscle at the expense of reduced exercise capacity that persisted for the duration of the study. These findings underscore the need for further comprehensive investigations into the mechanisms of obesity resistance, as they hold potential implications for weight-loss strategies in human subjects.

## 1. Introduction

The variety of individual responses to an obesogenic environment, the onset of obesity, and its direct relationship with various health outcomes are not fully understood [1]. It has been established that obesity, as defined by the body mass index (BMI), is heritable across a lifetime, with an overall effect of up to 70% [2]. However, within a specific environment, significant differences in body weight and fat mass among individuals imply that adiposity is additionally shaped by intricate interplays of other metabolic, behavioral, and environmental factors. Aside from the monogenic genetic aberrations in the central appetite regulatory pathway or the syndromic forms [3], obesity is highly polygenic in its nature and involves millions of common genetic variants, each having a small effect on an individual’s susceptibility to gaining weight [4]. Although genome-wide association studies (GWAS) were successful at identifying the *FTO* locus with a relatively large effect on BMI (1 kg of additional body weight for an average adult), the combined effects of this and 750 other minor GWAS loci known to date explain only 6% of variation [5]. Recent additional GWAS that focused on more refined obesity outcomes such as resistance to weight gain suggested that the characterization of obesity-resistant (non-responders) phenotypes may provide an alternative understanding of the metabolic complexity of body weight regulation [6].

Dietary fats are essential nutrients and a concentrated source of energy that serves various vital functions in the human body [7]. Consuming fats contributes to feelings of fullness and satisfaction, signaling to the brain that the body has received sufficient energy [8]. Unlike carbohydrates, which are stored in limited quantities as glycogen in the liver and muscles, fats can be stored in adipose tissue in virtually unlimited amounts [1]. While daily carbohydrate balance is tightly regulated so that the majority of carbohydrates are immediately and preferentially metabolized when available [9], dietary fats are primarily targeted for deposition and storage [10]. Insulin secreted in response to the carbohydrate load of a mixed meal further facilitates fat storage and decreases fat oxidation [11]. Previous rodent studies suggested that progressive metabolic abnormalities in response to calorie-dense meals follow the sequence of hyperinsulinemia; increased adipocyte size; greater adiposity; lower energy expenditure; and, eventually, increased hunger [12]. The excess energy is therefore deposited into conventional adipose depots, as well as ectopic non-target tissues like the liver and muscle based on the individual, hypothetical body weight set points, and patterns of fat accumulation [13].

A few previous animal studies were aimed at understanding the differences between the obesity-prone versus obesity-resistant phenotype, with limited success. Among the reported observations, the abilities of type I (slow) [14], type IIa (fast and oxidative) [15], and type IIb (fast and glycolytic) muscle fibers to alter nutrient partitioning for oxidation, upregulate of uncoupling proteins in the mitochondrial inner membrane of white and brown adipose tissue to increase dissipative heat [16], and decrease AMPK phosphorylation and PPARγ activity [17] have been suggested as putative aberrations without further mechanistic explanations. Several attempts at investigating gene pathways associated with lipid deposition in the adipose tissues were either inconclusive [18] or suggested several gene candidates, including *Acaca*, *Acly*, *Acss2*, *Aldh1a1*, *Elvol6*, *Scd1*, and *Slc25a1* [19], for further studies. Finally, serum profiles of obesity-resistant animals showed relative decreases in intermediates of the Krebs cycle (citrate, malate, and α-ketoglutarate) and ornithine; increases in glycine; and reduced amounts of catecholamine metabolites, including homovanillic acid, vanillylmandelic acid, and *p*-hydroxyphenylacetic acid, alongside increased citrate in urine [20]. However, the precise metabolic changes responsible for partial resistance to obesity after consuming large amounts of dietary fats have not been determined.

In this study, we utilized C57BL/6J mice as a well-recognized model of polygenic obesity [21] to analyze a biological phenotype of resistance to obesity (non-responders) after exposure to excessive dietary fats. Obesity-prone super-responders (SRs) and obesity-resistant non-responders (NRs) were compared to differentiate changes in body composition and energy metabolism. SR mice switched back to a low-calorie diet served as an additional control to assess the reversibility of the observed changes and pinpoint tissues that failed to recover their function upon returning to normal body weight. Finally, for the first time, we assessed changes in fecal microbiome profiles associated with these perturbations.

## 2. Materials and Methods

### 2.1. Animals and Diets

**Figure 1:**
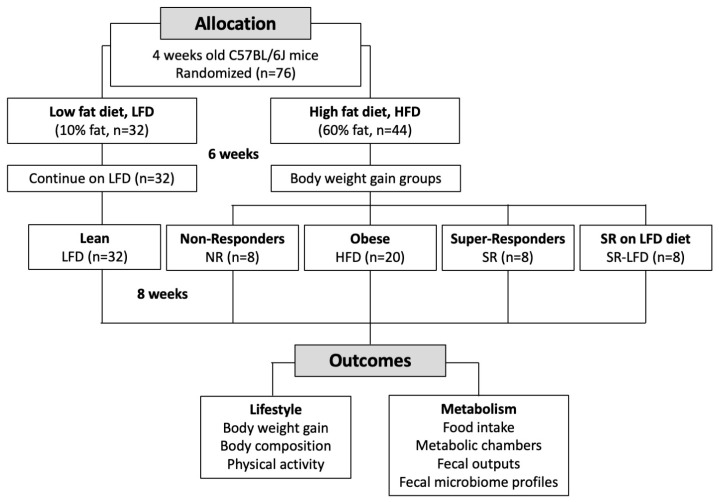
Flow chart of the study.

Male 4-week-old C57BL/6J mice were purchased from the Jackson Laboratory (Bar Harbor, ME, USA) and housed four animals per cage under controlled temperature (24 ± 2 °C) and light (12 h light–dark cycle, lights on at 7:00 a.m.) conditions. Immediately upon arrival, animals were allowed to adapt to the new conditions for 7 days, and the animals were handled daily to reduce the stress of physical manipulation. Mice were then randomized based on the initial body weights into ad libitum access to Research Diets (New Brunswick, NJ, USA) low-fat diet (LFD, D12450J, 10 kcal % fat, 3.85 kcal/g, n = 32) or high-fat diet (HFD, D12492, 60 kcal % fat, 5.24 kcal/g, n = 44) for 6 weeks. Obese animals were further randomized based on their body weights into normally obese (HFD), non-responders (NR), super-responders (SR), or super-responders switched back to the low-fat diet (SR-LFD) for an additional 8 weeks (Figure 1). The study was performed as a parallel arm with shared LFD and HFD controls [22]. All animal experiments were performed according to procedures approved by the NC Research Campus Institutional Animal Care and Use Committee in the David H. Murdock Research Institute (Kannapolis, NC, USA), an AAALAC-accredited animal care facility (protocol No. 12-018, approved on 10 April 2013).

### 2.2. Body Composition

Animal weight and food intake (accounting for spillage) were recorded weekly for the duration of the study. Body composition analysis was performed on unanesthetized mice using EchoMRI (Echo Medical Systems, Houston, TX, USA) during the last week of the study.

### 2.3. Energy Expenditure

An open-circuit LabMaster Metabolism Research Platform (TSE Systems, Bad Homburg, Germany) was used to assess indirect calorimetry and activity at the Animal Metabolism Phenotyping Core, UNC Nutrition Obesity Research Center. Rates of oxygen consumption (VO_2_) and carbon dioxide production (VCO_2_) were recorded in accordance with a reference cage every 12 min for 72 h. VCO_2_/VO_2_ was defined as the respiratory exchange ratio (RER), and energy expenditure (EE) was estimated using the equation = [3.815 + (1.232 × RER)] × VO_2_. The non-protein respiratory quotient table [23] was used to compute lipid and carbohydrate oxidation rates. The ActiMot system (TSE) was applied to measure activity by measuring infrared beam breaks in horizontal (x and y directions, running) and vertical (z direction, rearing) planes. All measurements were performed in individual animals.

### 2.4. Exercise Capacity

Endurance exercise capacity (run time) was determined on a rodent treadmill (Columbus Instruments, Columbus, OH, USA) with a shock grid set at <0.34 mA and 1 Hz. The treadmill intensity was set to 10 with a 0 incline. After 2 min of accommodation time, treadmill speed was set to 16 cm/s and progressively increased by 2 cm/s every 2 min until a maximum speed of 24 cm/s was reached. The test was terminated when animals received 3 consecutive electrical stimuli and failed to move, or 20 min passed.

At the end of the experiment, blood was collected into serum tubes by heart puncture after CO_2_ inhalation. Metabolic tissues (liver, fat, muscle, and brain) were collected and stored at −80 °C to determine the temporal sequence and signaling events that were responsible for changes in physiology and metabolism.

### 2.5. Fecal Parameters

Fecal pellets were collected over 1–2 consecutive days the end of the study. Fresh trays were gently removed, and pellets were collected in Eppendorf tubes using clean forceps. Pellets in contact with other surfaces were excluded. After collection, pellets were counted, weighed individually, and immediately frozen at −80 °C until analyzed.

Total fecal lipids were extracted from pre-weighed fecal pellets using the Folch method [24]. The pellets were homogenized with chloroform/methanol (2/1) to a final volume of 20 times the weight of the sample and incubated at room temperature for 2 h. Samples were then centrifuged at 3000× *g* for 10 min, and the supernatant was mixed with an equal amount of 0.9% NaCl solution. Following a second centrifugation, the upper aqueous phase was discarded, and the lower chloroform phase containing the fat was evaporated using a Rotavapor R210 (Buchi Labortechnik, Flawil, Switzerland) under vacuum. The lipid weight was determined as a percentage of the wet fecal pellet weight.

### 2.6. Fecal Microbiome Profile

Genomic DNA was extracted from mouse fecal samples using QIAamp Fast DNA Stool Mini kits (Qiagen, Germantown, MD, USA), quantified using a Take3 plate and Synergy H1 microplate spectrophotometer (BioTek, Sunnyvale, CA, USA), and adjusted to a final concentration of 1 ng/μL. Quantitative real-time PCR was performed on an ABI 7500 Fast instrument (Life Technologies, Carlsbad, CA, USA) in a total volume of 20 μL containing 10 μL 2× SYBR Green PCR Master Mix, 1 μL of each primer from the GUt Low-Density Array (GULDA) [25], 4.4 μL of nuclease-free water, and 3.6 μL of template DNA. The amplification program consisted of 50 °C for 2 min; 95 °C for 10 min; and 40 cycles of 95 °C for 15 s and 60 °C for 1 min. A dissociation curve was recorded at 95 °C for 15 s and 60 °C for 15 s; then, the temperature was increased to 95 °C at a 2% rate). The mean Ct value was determined based on a set threshold value of 0.2 and using automatic baseline correction. Differences in Ct values for each bacterial target (N0 normalization) were calculated between those obtained with the universal and target-specific primers and log-transformed. Fold changes for target amplicons were calculated as the (log 2) ratio of normalized abundances and determined as a percentage of the microbiome composition.

### 2.7. Statistical Analysis

Data were analyzed by one-way ANOVA followed by Dunnett’s multiple-range tests using Prism 8.0 (GraphPad Software, San Diego, CA, USA). Temporal measures were analyzed by two-factor repeated-measures ANOVA, with time and treatment as independent variables. All data were presented as means ± SEM. Significant differences were accepted when the *p*-value was <0.05.

## 3. Results

### 3.1. Changes in Body Weight and Body Weight Gain

At the beginning of the study, 4-week-old C57BL/6J mice (n = 76) were randomly assigned to either LFD (n = 32) or HFD (n = 44) ad libitum for 6 weeks. During this initial period, there was no significant difference in food intake between HFD and LFD mice; however, the increased caloric density of the HFD (5.24 versus 3.85 kcal/g, respectively) allowed for early induction of obesity in the HFD group.

C57BL/6J mice, as a polygenic developmental model of diet-induced obesity [21], allowed us to select the HFD animals differing in body weight and fat accumulation despite their inbred genetic background. Mice highly susceptible to the development of obesity that reached body weights in excess of 36 g after being fed an HFD for 6 weeks were designated as obesity-prone or super-responders (SR, n = 16). These animals were further divided into two equal groups, one of which was switched back to LFD treatment for the duration of the study (SR-LFD, n = 8). On the other hand, mice that failed to cross the 30 g body weight threshold under similar dietary conditions were designated as obesity-resistant or non-responders (NR, n = 8). Control animals fed the corresponding LFD or HFD reached average body weights of 26.2 ± 2.3 and 33.1 ± 3.1 g in the same timeline, respectively.

**Figure 2:**
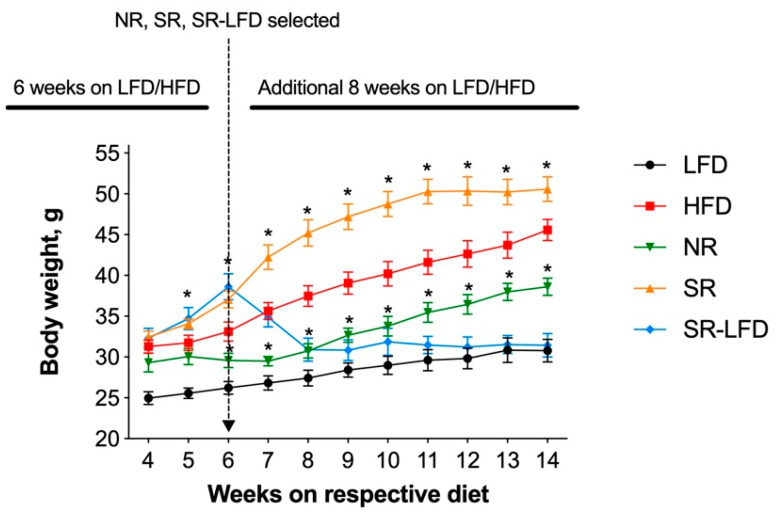
Body weight of lean controls (LFD), obese controls (HFD), obesity-resistant non-responders (NR), obesity-prone super-responders (SR), and super-responders an LFD during the study −14 (SR-LFD). Results are expressed as means ± SEM (n = 8). Data were analyzed using a two-factor repeated-measures ANOVA; * *p* < 0.05 versus the HFD controls.

All mice were kept on their respective diets for an additional 8 weeks. This allowed us to differentiate and further amplify the individual variations in response to the obesogenic dietary fats. Control LFD animals continued a slow, gradual increase in body weight and reached an average body weight of 30.8 ± 3.9 g at the end of the study. The HFD controls reached 45.6 ± 3.5 g body weights (*p* < 0.05). Obesity-prone SR mice rapidly gained excessive body weight and plateaued at an average of 50.6 ± 3.0 g for the last 4 weeks of the study. Obesity-resistant NR mice failed to gain body weight in excess of 38.6 ± 2.1 g, a −15.4% decrease relative to the HFD controls and a −23.7% decrease relative to the SR mice. Obesity-prone SR-LFD mice fed the LFD rapidly lost weight within 3 weeks of the dietary change, and their body weights remained undistinguished from the LFD controls at the end of the study (Figure 2).

**Figure 3:**
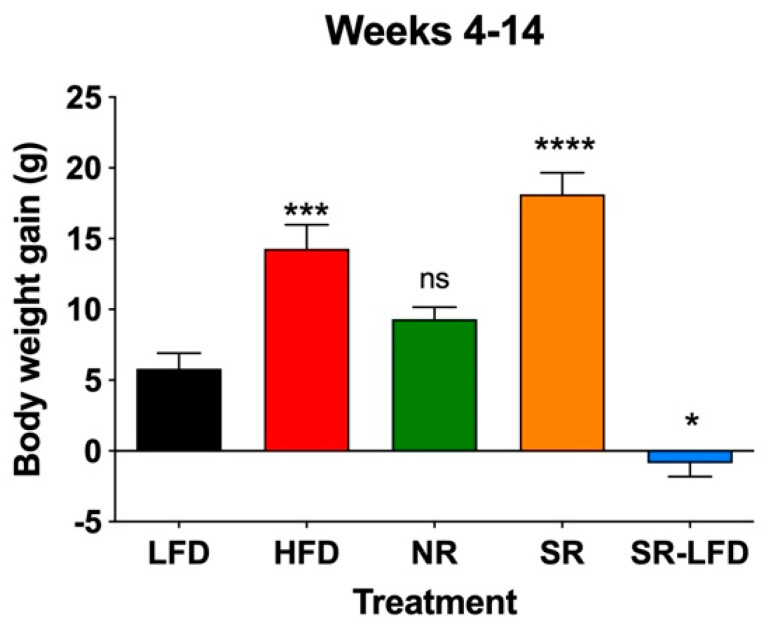
Body weight gain of lean controls (LFD), obese controls (HFD), obesity-resistant non-responders (NR), obesity-prone super-responders (SR), and super-responders fed an LFD during the study −14 (SR-LFD). Results are expressed as means ± SEM (n = 8). Data were analyzed using one-way ANOVA followed by Dunnett’s multiple comparisons; * *p* < 0.05, *** *p* < 0.001, **** *p* < 0.00001, and ns (not significant) versus the LFD controls.

When body weight gains were determined for study weeks 4–14, SR mice gained 18.1 ± 4.5 g, corresponding to an excess of 20.9% relative to the HFD controls (14.3 ± 4.5 g). NR mice showed reduced body weight gain of 9.3 ± 1.7 g, despite no statistically significant difference relative to the LFD controls (5.8 ± 3.2 g). When compared directly, SR mice gained 48.6% more body weight than their NR counterparts, highlighting the individual differences despite the shared C57BL/6J background and identical HFD. The SR-LFD animals that returned to the LFD for the last 8 weeks of the study showed negative body weight gain (−0.9 ± 1.9), essentially losing additional body weight accumulated during the first part of the study (Figure 3).

### 3.2. Changes in Body Composition

**Figure 4:**
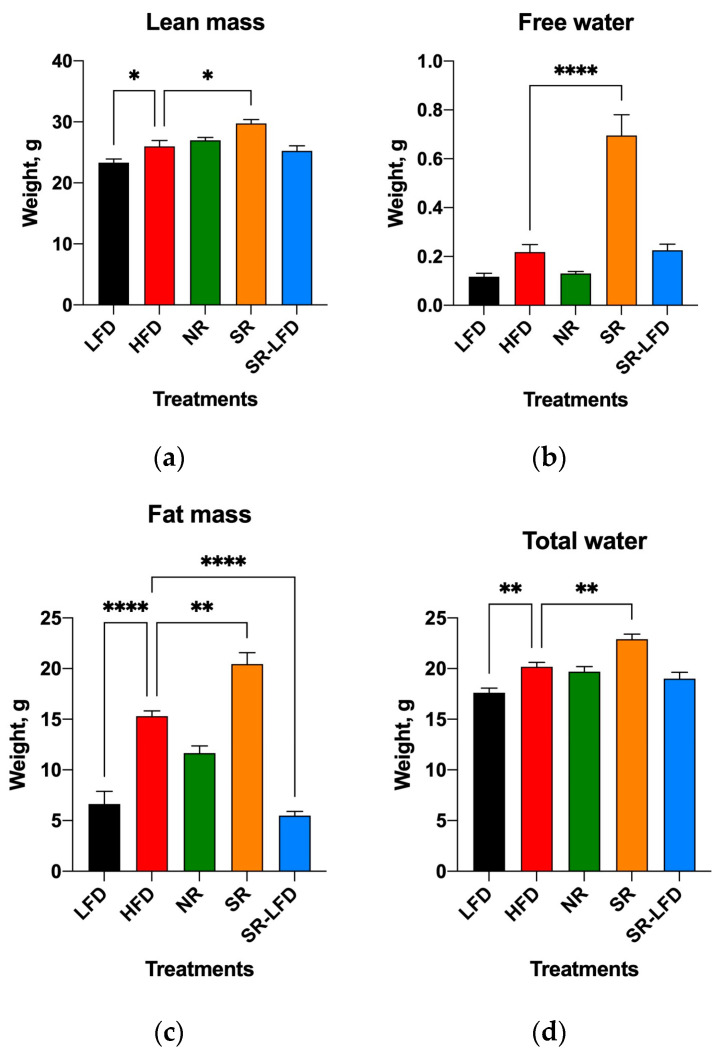
Body composition of lean controls (LFD), obese controls (HFD), obesity-resistant non-responders (NR), obesity-prone super-responders (SR), and super-responders fed an LFD, including (**a**) lean body mass, (**b**) free water, (**c**) fat body mass, and (**d**) total water. Results are expressed as means ± SEM (n = 8). Data were analyzed using one-way ANOVA followed by Dunnett’s multiple comparisons; * *p* < 0.05, ** *p* < 0.01, and **** *p* < 0.00001 versus the HFD controls.

Body weight gains correlated with significant changes in body composition in all groups at the end of the study. As expected, the mean body weights of HFD mice increased by 59.4% relative to the LFD controls, and this increase consisted of a 11.5% increase in lean body mass and a 56.8% increase in fat body mass due to the high-fat nature of the HFD (Figure 4a,c). Similar to HFD mice, NR animals fed an HFD gained 13.6% more lean body weight, despite an only 43.2% increase in fat body mass, indicating changes in the skeletal muscle tissues and reduced development of adipose depots under the same feeding conditions. SR mice gained 21.6% more lean body mass and 67.7% more fat body mass than the LFD controls, corresponding to a 25.2% increase in fat body mass relative to normally obese HFD mice (*p* < 0.01, Figure 4c). The SR-LFD animals that returned to the LFD showed both lean and fat body masses that returned to the levels of the LFD controls.

However, SR mice showed a dramatic increase in free water that exceeded that of the HFD controls by a factor of 3.1 fold and that of the LFD controls by a factor of 5.8 fold. NR mice showed free water content equal to that of the LFD controls (Figure 4b). This was observed in the background of marginally increased total body water (1.3 fold relative to LFD mice and 1.1 fold relative to HFD mice), which cannot explain the difference in free water that is generally associated with bladder content (Figure 4d).

### 3.3. Metabolic Responses to Dietary Fat

**Figure 5:**
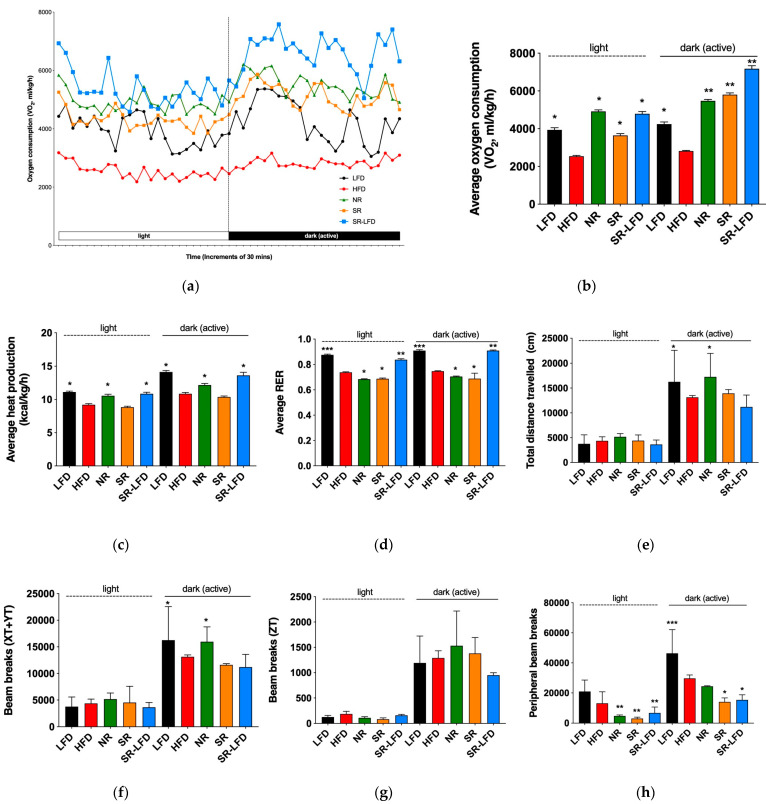
Changes in whole-body energy balance in response to dietary fats as compared among lean controls (LFD), obese controls (HFD), obesity-resistant non-responders (NR), obesity-prone super-responders (SR), and super-responders fed an LFD. Indirect calorimetry was used to determine (**a**) the dynamics of oxygen consumption; (**b**) average oxygen consumption; (**c**) average heat production; (**d**) average RER; (**e**) distance traveled; (**f**) voluntary physical activity in the horizontal plane; (**g**) voluntary rearing; and (**h**) fine, agitation-like movements. Results are expressed as means ± SEM (n = 8). Data were analyzed using one-way ANOVA followed by Dunnett’s multiple comparisons; * *p* < 0.05, ** *p* < 0.01, and *** *p* < 0.001 versus the HFD controls.

We next used open-circuit indirect calorimetry to measure energy expenditure, including VO_2_, VCO_2_, RER, and voluntary physical activity using infrared beam breaks in the horizontal and vertical planes (Figure 5). All metabolic parameters related to energy metabolism were normalized to lean body mass to account for differences in animal body weights, as per standard considerations [26].

The dynamics of oxygen consumption during light (inactive) and dark (active) cycles are shown in Figure 5a and averaged in Figure 5b. As expected, HFD mice showed decreased oxygen consumption relative to LFD controls in both cycles, with mean VO_2_ values of 2542 ± 300 and 2815 ± 269 mL/kg/h, respectively. Both SR and NR mice showed elevated oxygen consumption during the active cycle, but only NR mice maintained high oxygen consumption levels during the inactive cycle (4918 ± 548 mL/kg/h, *p* < 0.05). This subsequently translated to higher levels of heat production during both the inactive (10.6 ± 1.6 kcal/kg/h, *p* < 0.05) and active (12.2 ± 1.7 kcal/kg/h, *p* < 0.05) cycles. SR mice failed to upregulate heat production in both cycles, which serves as indirect evidence of inefficient energy metabolism, despite increased oxygen consumption during the active cycle (Figure 5c).

All animal groups fed an HFD showed respiratory exchange ratios (RERs) in the range of 0.685–0.748, indicating that they all relied on dietary fats as primary metabolic fuel. The lowest mean RER value was observed in NR mice during the inactive cycle (0.685 ± 0.022, *p* < 0.05), suggesting an upregulated lipid metabolism despite relative inactivity at rest (Figure 5d).

Voluntary physical activity was increased in NR mice and reached the basal levels of the LFD controls in the active cycle both in terms of total distance traveled (Figure 5e) and horizontal plane movements (Figure 5f) but not vertical rearing (Figure 5g). Peripheral beam breaks that capture fine, agitation-like movements were reduced (Figure 5h).

SR-LFD mice fed an LFD showed clear signs of increased energy expenditure (Figure 5b,c) and a fuel shift towards utilization of carbohydrates (Figure 5d) that matched the body weight loss observed after the dietary switch. However, their voluntary physical activity remained at the level of SR mice and did not improve with time (Figure 5e,h).

### 3.4. Irreversible Changes in Exercise Capacity

**Figure 6:**
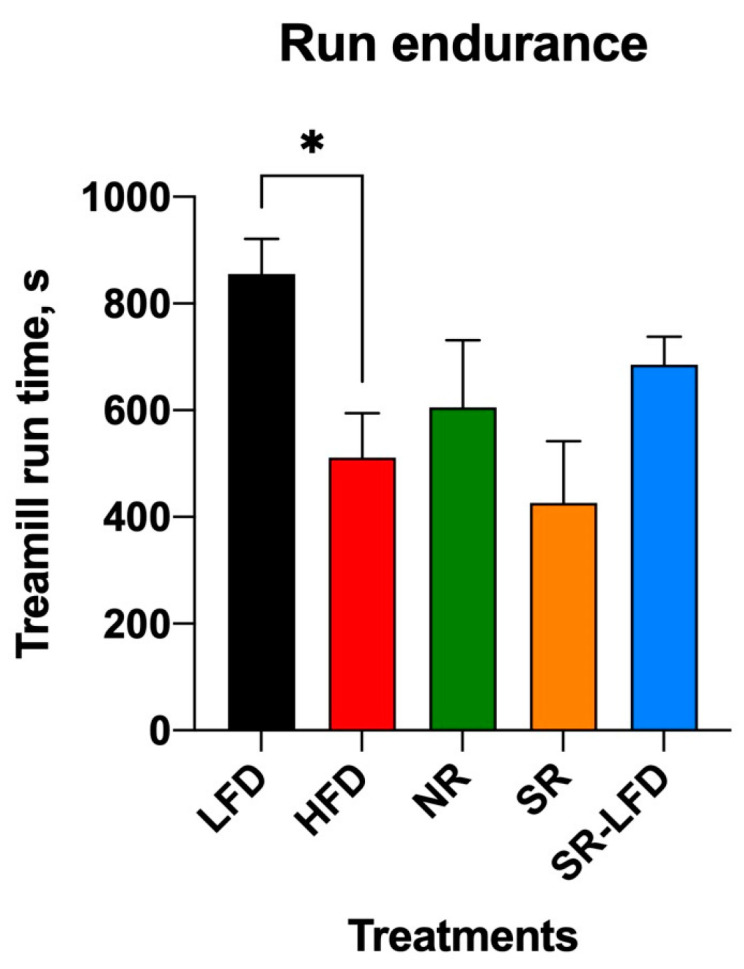
Exercise capacity of lean controls (LFD), obese controls (HFD), obesity-resistant non-responders (NR), obesity-prone super-responders (SR), and super-responders fed an LFD at the end of the study. Results are expressed as means ± SEM (n = 8). Data were analyzed using one-way ANOVA followed by Dunnett’s multiple comparisons; * *p* < 0.05 versus the HFD controls.

Obese HFD, SR, and SR-LFD mice showed decreased voluntary physical activity that can be partially explained by excessive body weight. The inability of SR-LFD mice to restore their voluntary physical activity to the basal levels (Figure 5e,h), despite the switch to the LFD and the corresponding weight loss (Figure 2), prompted us to evaluate changes in their exercise capacity using a rodent treadmill. Similar to the metabolic chamber data, obese HFD controls showed decreased exercise capacity in a running exercise on a treadmill (511 ± 204 s versus 856 ± 160 s for the LFD controls, *p* < 0.05) (Figure 6).

Notably, both NR mice and SR-LFD mice that lost excessive body weight after a dietary switch to the LFD failed to alter or regain the basal exercise capacity, despite significantly lower body weights (Figure 2) and comparable lean body mass (Figure 4a) at the end of the study. These data suggest that detrimental effects induced by high levels dietary fats failed to resolve even after animals returned to the LFD, at least within the timeline of this study (8 weeks).

### 3.5. Food Intake and Feed Efficiency

**Figure 7:**
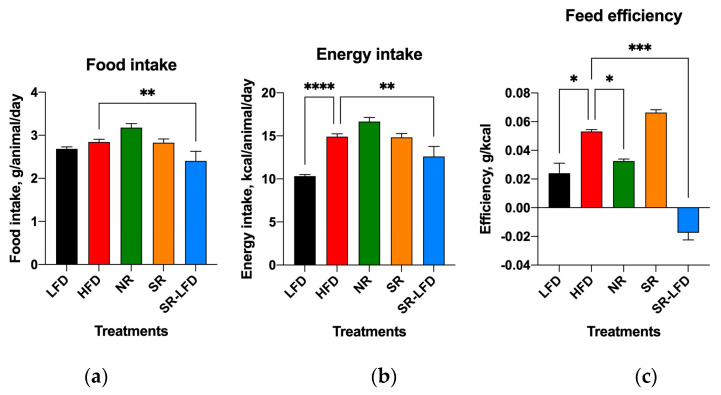
Changes in (**a**) food intake, (**b**) energy intake, and (**c**) feed efficiency among lean controls (LFD), obese controls (HFD), obesity-resistant non-responders (NR), obesity-prone super-responders (SR), and super-responders fed an LFD. Results are expressed as means ± SEM (n = 8). Data were analyzed using one-way ANOVA followed by Dunnett’s multiple comparisons; * *p* < 0.05, ** *p* < 0.01, *** *p* < 0.001, and **** *p* < 0.00001 versus the HFD controls.

Control animals consumed, on average, 2.68 ± 0.21 g per LFD animal per day and 2.84 ± 0.29 g per HFD animal per day for the duration of the study (Figure 7a). Although daily food intakes were similar between LFD and HFD animals, the caloric densities of these diets differed significantly (3.85 kcal/g versus 5.24 kcal/g). Therefore, HFD mice experienced larger energy intakes in the range of 14.88 kcal/animal/day versus 10.32 kcal/animal/day for the LFD counterparts. Nearly all additional calories came in the form of dietary fats due to the nature of the HFD.

Both SR mice and NR mice showed average food intakes similar to those of HFD controls, suggesting that differences in energy intakes were not responsible for the observed changes in body weight gain and body composition (Figure 7a,b). If anything, NR mice showed increased food intake that nearly reached statistical significance (3.18 ± 0.28 g per animal per day, *p* = 0.066). Their feed efficiency—or the ability to convert consumed food into a body weight gain—was reduced by 39.6% (0.033 ± 0.002 g/kcal versus 0.053 ± 0.002 g/kcal for HFD controls, *p* = 0.043). SR-LFD mice showed negative feed efficiency due to progressive body weight loss after switching from an HFD to the LFD (Figure 7c).

### 3.6. Fecal Lipids and Microbiome Profiles

**Figure 8:**
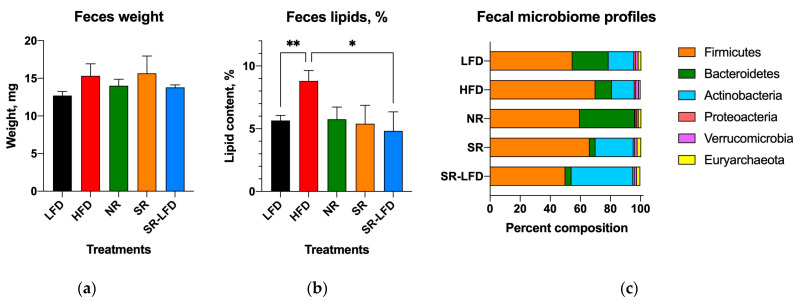
Fecal outputs and microbiome profiles determined at the end of the study among lean controls (LFD), obese controls (HFD), obesity-resistant non-responders (NR), obesity-prone super-responders (SR), and super-responders fed an LFD, including (**a**) fecal pellet weight, (**b**) fecal fat content, and (**c**) microbiome composition at the phylum level. Results are expressed as means ± SEM (n = 8). Data were analyzed using one-way ANOVA followed by Dunnett’s multiple comparisons; * *p* < 0.05 and ** *p* < 0.01 versus the HFD controls.

We also quantified fecal output and fecal lipid content to account for discrepancies between intake and metabolism of dietary fat in SR and NR mice. There were no changes in fecal pellet characteristics among any of the study groups (Figure 8a). However, the lipid content of the feces was higher for HFD controls (8.8 ± 2.9%) than the LFD controls (5.7 ± 1.4%, *p* = 0.008). Unexpectedly, both SR and NR mice showed lipid fecal contents similar to the LFD controls (Figure 8b), suggesting that both groups absorbed dietary lipids more efficiently than the HFD mice, yet they processed and/or metabolized them differently, which resulted in body weight gain and fat body mass disparities among these groups.

Fecal microbial profiles were strongly affected by the obesity-prone and obesity-resistant phenotypes. The LFD controls showed a typical murine fecal microbiome profile consisting of 55% *Firmicutes*, 24% *Bacteroidetes*, 17% *Actinobacteria*, 1.8% *Verrucomicrobia*, 1.8% *Euryarchaeota*, and 1% *Proteobacteria*. Feeding with an HFD resulted in subtle shifts in the fecal microbiome profile towards increased *Firmicutes* (70%) and decreased *Bacteroidetes* (11%). These changes were potentiated in the SR obesity-prone mice, with a further reduction in *Bacteroidetes* (4%) and expansion of *Actinobacteria* (25%). The NR obesity-resistant mice showed a clear opposing trend, with expansion of *Bacteroidetes* (37%) and a reduction in *Actinobacteria* (1%). Even after switching to the LFD, SR-LFD mice preserved the obesity-prone fecal microbiome profile, with reduced *Bacteroidetes* (4%) and expanded *Actinobacteria* (41%) until the end of the study (Figure 8c). Unfortunately, the lack of prestudy fecal samples prevented us from drawing conclusions about the initial microbial profiles in these animals.

## 4. Discussion

Individual metabolic variations can significantly influence predisposition to obesity, especially in the face of contemporary dietary habits [27]. Human bodies metabolize nutrients differently based on a multiplicity of factors, influencing how efficiently dietary fats, carbohydrates, and proteins are processed. This inherent diversity means that some individuals might have a lower metabolic efficiency and a higher propensity to store excess calories as fat, making them more susceptible to weight gain [28]. When these metabolic differences intersect with the prevalence of modern processed foods, the impact on obesity rates becomes significant. Calorie-dense yet nutritionally poor options can lead to weight gain, particularly when combined with larger meal sizes, which have become the norm in many societies. Additional calories not only contribute to weight gain but also pose serious health risks, impacting cardiovascular health and further elevating the risk of obesity-related complications [29]. Moreover, individuals prone to fat accumulation and obesity face additional hidden malnutrition challenges through acute and chronic metabolic disorders that are often present in overweight and obese states [30]. This is particularly true for conditions that include insulin resistance, ectopic fat accumulation, and mitochondrial disfunction [31].

As animals were fed an HFD with an excess of dietary fats during the first 6 weeks of the study, they gained body weight and adjusted their body composition to accommodate for additional energy intake. Due to the polygenic nature of C57BL/6J obesity, there was a significant variation in this process that allowed us to select obesity-prone super-responders in the upper quartile (SR) and obesity-resistant non-responders (NR) in the lower quartile, similar to previous studies [14,16,17,19]. The greatest body weight gains observed in SR mice were accompanied by increased in lean body mass, fat body mass, and total and free water. The last measure is peculiar because it largely determines free urine in the bladder. This seems to indicate that SR mice attempted to counteract excessive dietary fat by increasing their water intake [32] (which was not measured in this study) or required more water to suppress whole-body lipolysis [33]. Otherwise, SR mice had food intake, fecal output, and RER quotients similar to those of NR mice, suggesting that differences in energy metabolism were the primary reason behind the observed individual variation.

Some of the energy expenditure data hinted that target tissues may be responsible for this difference. NR mice showed higher oxygen consumption during the light (rest) cycle of the day, suggesting that their resting metabolic rate (RMR) was increased. RMR is one of the major contributors to weight stability, resulting predominantly from the thermic effects of skeletal muscles (including heart) and the digestive tract [34]. Average heat production was also increased in NR animals, as was voluntary physical activity, similar to what was observed in previous studies [16,19]. There might not be a single ideal RMR, given the impact of food availability in natural settings that are highly variable; however overall reproductive success in mammals is often positively correlated with resting metabolic rate [35]. As NR mice consumed, on average, 12% more of the HFD, excreted similar amounts of lipids in feces, and accumulated 75.6% less fat body mass than SR animals, the net balance of the remaining lipids requires increased capacity for their metabolism normally achieved by the metabolic flexibility of the skeletal muscle [36]. Shifting towards increased reliance on fat oxidation may conserve the use of plasma glucose and postpone the depletion of muscle glycogen under the conditions of high dietary fat intake (by elevated plasma fatty acids) or after fasting when food is not available. As such, diminished metabolic flexibility of skeletal muscle to oxidize fat under conditions of low carbohydrate and high dietary fat load is strongly predictive of weight gain [37].

Although there is considerable interest in understanding the adaptive significance of variations in metabolic rates, there have been surprisingly few empirical efforts to test whether there is a correlation between metabolic rates and individual fitness—or at least components of fitness. This study evaluated the exercise capacity of both NR and SR mice in comparison with control animals and showed a 29.2–50.2% decrease as compared to healthy, lean animals but only 15.5–16.6% variation when compared to the obese controls. These findings suggest that metabolic flexibility of the skeletal muscle to increase fat oxidation is achieved at the expense of muscle function. This is especially highlighted in SR-LFD mice that were fed a low-fat diet during the last 8 weeks of the study, which showed a near-complete return to healthy body weight, lean and fat body mass, and increased energy metabolism that persisted to sustain the weight loss. However, despite returning to the normal body weight, SR-LFD mice failed to regain an exercise capacity similar to that of control animals, once again suggesting an irreversible change in the skeletal muscle function that was resolved for the duration of the study.

The contribution of microbial enterotypes to individual nutrition and obesity management remains largely unexplored [38]. It is well established that out of thousands of bacterial species found in the gut, the majority belong to two phyla, i.e., *Firmicutes* and *Bacteroidetes*, which encompass ~90% of the microbiota; the minimal model microbiome additionally contains *Proteobacteria*, *Actinobacteria*, *Fusobacteria*, *Verrucomicrobia*, and *Euryarchaeota* [39]. Microbial enterotypes can be polarized based on the predominant macronutrients and energy substrates in the diet [40], as seen for *Bacteroidetes*, which can be dominated by the *Bacteroides* genus in association with diets high in animal protein and fat, or by the *Prevotella* genus in diets rich in plant fiber [41]. Some studies have reported that major enterotypes remain largely unchanged, even after 6 months of dietary adjustments [42]. The stability of the less prominent phyla is poorly understood, for example, *Actinobacteria* seem to dominate the gastrointestinal tracts of young children due to consumption of human milk, but plummet to a minor status shortly after weaning [43]. In this study, we observed that the *Bacteroidetes* phylum was expanded in obesity-resistant NR mice at the expense of the *Actinobacteria* phylum, which was considerably diminished. The significance of this finding remains to be elucidated, as it was previously reported that enriching the gut microbiome with *Actinobacteria* (specifically, the *Bifidobacterium* genus) with oligofructose prebiotics positively correlated with improved glucose tolerance and insulin secretion [44].

## 5. Conclusions

Together, these findings underscore the significant role of individual metabolic variations in influencing susceptibility to obesity in the context of contemporary dietary habits and modern centralized agricultural production systems [45]. The inherent diversity in nutrient utilization contributes to differences in metabolic efficiency, impacting the storage of excess calories as fat and increasing the vulnerability to weight gain. Differences in energy metabolism in the form of resting metabolic rate, skeletal muscle metabolic flexibility, and weight stability suggest a tradeoff between supporting high rates of fat metabolism and maintaining healthy muscle function.

Furthermore, this study draws attention to the unexplored contribution of microbial enterotypes to individual nutrition and obesity management, emphasizing the need for further investigation of the stability of gut microbiome composition in response to dietary adjustments. The major divergence in obesity phenotypes, energy metabolism, fecal microbiome profiles, and functional status of skeletal muscles in subjects prone to or resistant to developing obesity warrants a critical new look at biochemistry and gene expression pathways in the muscle tissues that are responsible for these effects.

## Data Availability

Data are contained within the article.
